# eDNA Metabarcoding Reveals Diel Connectivity Dynamics of Fish Communities in Xincun Lagoon, Hainan

**DOI:** 10.3390/ani16142268

**Published:** 2026-07-22

**Authors:** Jinfa Zhao, Hong Li, Teng Wang, Yong Liu, Juan Shi, Peng Wu, Yayuan Xiao, Jian Zou, Yu Liu, Lin Lin

**Affiliations:** 1 College of Fisheries, Hunan Agricultural University, Changsha 410128, China; zhaojf2026@hunau.edu.cn (J.Z.); lihongfish@hunau.edu.cn (H.L.); 2 Scientific Observation and Research Station of Xisha Island Reef Fishery Ecosystem of Hainan Province, Sanya Tropical Fisheries Research Institute, Sanya 572018, China; wt3074589@163.com (T.W.); liuyong@scsfri.ac.cn (Y.L.); sjuan0917@163.com (J.S.); wupeng@scsfri.ac.cn (P.W.);; 3 Key Laboratory of South China Sea Fishery Resources Exploitation & Utilization, Ministry of Agriculture and Rural Affairs, South China Sea Fisheries Research Institute, Chinese Academy of Fishery Science, Guangzhou 510300, China

**Keywords:** eDNA metabarcoding, lagoon, diel dynamics, ecological corridor, fish connectivity, Clupeidae

## Abstract

Coastal lagoons are crucial transitional zones connecting land and sea, and their ecological connectivity with adjacent open waters directly sustains regional biodiversity and ecosystem functions. However, how lagoon fish communities connect on a diurnal timescale remains poorly understood. In this study, we selected Xincun Lagoon on the southeastern coast of Hainan Island, China, as the study area. We established six sampling stations along an environmental gradient from the lagoon interior, through the tidal inlet, to the open sea, and conducted repeated diurnal sampling at six time points on 24 January 2024. Using eDNA metabarcoding, we analyzed the spatiotemporal dynamics of fish communities to examine spatial functional differentiation within the lagoon ecosystem and the potential corridor role of the tidal inlet. Our results showed clear spatial functional differentiation of fish communities along the environmental gradient. The lagoon inlet (XC4) exhibited the highest rate of community temporal change and the greatest amplitude of diversity fluctuation, with a peak change rate of 0.854 during the evening period (18:00–21:00). Its coefficient of variation for Shannon diversity was 6.2 times that of the mangrove creek (XC3). The mangrove creek area (XC3) had the highest Shannon diversity and the smallest fluctuations, functioning as a biodiversity hotspot and a region of relatively stable community structure. Within the lagoon interior, the temporal dynamics at the marginal zone (XC1) were substantially stronger than those in the central zone (XC2). Offshore stations indicated that the ecological influence of the lagoon extended to the 5 m isobath but weakened at the 10 m isobath. Furthermore, Clupeidae eDNA signals were strongest inside the lagoon during the day, at the tidal inlet in the evening, and in offshore waters at night. This study demonstrates the utility of eDNA metabarcoding for capturing fine-scale diel variation in fish community composition and distribution across the lagoon–inlet–offshore gradient, providing information that is difficult to obtain through conventional surveys. These patterns suggest, rather than confirm, that the tidal inlet may play an important role as a corridor linking lagoon and offshore habitats, and provide a reference case for using eDNA technology to study fine-scale connectivity in similar systems.

## Simple Summary

Coastal lagoons support many fish species that move between inshore and offshore waters, but daily movement patterns through tidal inlets remain poorly understood. In this study, we used environmental DNA (eDNA) technology to detect diel variation in fish community composition in Xincun Lagoon, Hainan Island, China, by collecting water samples at six locations and six time points from early morning to night. Fish diversity was highest and most stable in the mangrove creek, while the tidal inlet showed the strongest evening community shifts. Clupeidae showed eDNA-based evidence suggesting diel movement: they were detected inside the lagoon during the day, at the inlet in the evening, and in offshore waters at night. These eDNA-based patterns are consistent with the inference that the tidal inlet may serve as a passageway for diurnal fish movement between the lagoon and the sea, providing information that is difficult to obtain through conventional surveys.

## 1. Introduction

Coastal lagoons are among the most productive and ecologically valuable ecosystems on Earth, playing important roles in nutrient cycling, fishery production, coastal protection, and biodiversity maintenance [1,2]. Located at the land–sea interface, their limited connectivity with open waters directly determines their hydrological regimes, sedimentary dynamics, and ecological community structure [3,4]. Globally, coastal lagoons provide crucial nursery habitats for numerous fish and invertebrate species, with many species depending on lagoons as shelter and feeding grounds during early life stages before migrating to nearshore or open waters [5,6]. This bidirectional movement between lagoons and the ocean constitutes an important ecological process—habitat connectivity—which underpins population replenishment, gene flow, and ecosystem resilience [7,8]. However, although the importance of connectivity for maintaining lagoon ecosystem structure and function is widely recognized, research has focused primarily on spatial patterns or seasonal changes. Fine-scale temporal dynamics of fish movement, especially on a diurnal scale along the gradient from lagoon through tidal inlet to open sea, remain relatively understudied.

Ecological corridors are spatially defined areas that maintain or restore ecological connectivity, a concept increasingly recognized in conservation science [9]. In marine systems, the theoretical development and practical application of ecological corridors lag behind those on land, mainly because the fluid and three-dimensional nature of the marine environment makes habitat boundaries more diffuse and hydrodynamic processes largely mediate organism dispersal [1,10]. In lagoon systems, the narrow tidal inlet connecting the enclosed water body with the open sea represents a natural example of a marine ecological corridor [1,11]. Nevertheless, empirical data showing how such corridors operate on a daily basis remain scarce.

The rapid development of environmental DNA (eDNA) metabarcoding has substantially improved our ability to monitor fish biodiversity along complex environmental gradients at high spatiotemporal resolution [12,13]. This technique detects trace genetic material released by organisms into water, enabling non-invasive multi-species detection [14,15]. Traditional monitoring methods—such as trawling, gillnetting, and underwater visual census—often suffer from gear selectivity, habitat accessibility issues, and difficulties in detecting cryptic or rare species. In contrast, eDNA offers an efficient alternative for biodiversity assessment [16,17]. Previous studies have confirmed that eDNA metabarcoding can capture broader fish community signals than conventional surveys in complex coastal and transitional ecosystems [18,19]. Most eDNA studies in coastal systems have focused on spatial comparisons [20], seasonal variation [21], or methodological validation [18]. Few studies have combined diurnal temporal sampling with a lagoon–inlet–offshore gradient to examine fine-scale connectivity signals, despite many fishes exhibiting well-known diurnal activity rhythms [22,23].

Therefore, we selected Xincun Lagoon on the southeastern coast of Hainan Island, China, as a natural laboratory to study diurnal-scale lagoon–ocean connectivity. This lagoon contains heterogeneous habitat types, including mangrove creeks, shallow tidal flats, and deeper central waters, collectively supporting a rich fish fauna typical of tropical Indo-Pacific nearshore ecosystems. We tested three specific hypotheses. First, fish community structure changes systematically along the lagoon–inlet–open sea gradient, with different functional zones characterized by distinct community assemblages. Second, the narrow tidal inlet shows the greatest diurnal variability in fish community composition, suggesting a possible corridor role in lagoon–sea connectivity. Third, the greatest temporal variation in fish communities occurs in the tidal inlet area.

## 2. Materials and Methods

### 2.1. Sample Collection and Processing

Lingshui Xincun Lagoon and its adjacent open sea (18°23′–18°27′ N, 109°56′–110°2′ E) are located in Xincun Town, Lingshui Li Autonomous County, on the southeastern coast of Hainan Island. This area features diverse habitat types and concentrated human activities, making it an ideal site for studying marine biological connectivity. Xincun Lagoon is approximately 4 km north–south and 6 km east–west, covering about 24 km^2^. It is surrounded by the Nanwan Peninsula, with only one narrow inlet approximately 150 m wide connecting it to the open sea; surface runoff input is minimal. Several irregular shoals and three mangrove stands are distributed within the lagoon.

Sampling was performed on 24 January 2024, during the low-tide slack-water period of the dry season in the study area, when tidal conditions were highly stable and environmental disturbances (including precipitation, atmospheric perturbations, tidal fluctuations, and surface runoff) were at minimal levels. This sampling strategy minimized the impact of external environmental fluctuations on the representativeness of water samples and eDNA detection results, thereby ensuring the comparability of samples collected across different time points. Replicate water samples were collected at each of six time points (R1–R6: 06:00, 09:00, 12:00, 15:00, 18:00, and 21:00) from six sampling sites (XC1–XC6) sequentially established along the lagoon interior-tidal channel-offshore axis (Figure 5). These six stations formed a complete environmental gradient from enclosed waters to the open ocean (Table 1). Tidal data for Xincun Bay on 24 January 2024 were obtained from the National Marine Data Center (http://mds.nmdis.org.cn/, accessed on 16 July 2026).

The entire sampling process at each time point was completed within 30 min to ensure high temporal consistency among samples. At each station, we collected 5 L of surface water using a peristaltic pump. To prevent cross-contamination, we used new pump tubing and sterile water collection bags for each station and thoroughly rinsed the tubing and containers with the water to be sampled before formal sampling. After collection, water samples were immediately stored at 4 °C. To prevent exogenous and inter-station DNA contamination, all equipment and consumables were soaked in 10% NaClO solution and heat-dried before boarding; before each station sampling, we thoroughly rinsed the equipment with water from that station. Samples were processed immediately on board using a vacuum filtration system: water was filtered through polycarbonate membranes (47 mm diameter, 0.2 µm pore size; Merck, Darmstadt, Germany), with three replicates per station (1.5 L per filter). After filtration, membranes were placed in 2 mL cryotubes and stored in liquid nitrogen at −196 °C. The vacuum filtration device was rinsed three times before and after processing each sample, and operators changed gloves when handling different samples. In addition, for each batch of samples, we filtered an equivalent volume of ultrapure water on the same day using the same equipment and procedures as a negative control. All sampling and filtration procedures strictly followed aseptic techniques to ensure the integrity of eDNA samples and prevent cross-contamination.

### 2.2. DNA Extraction and 12S rRNA Gene Amplicon Sequencing

We extracted filter membrane DNA using PowerWater DNA Isolation Kits (Qiagen, Hilden, Germany) following the manufacturer’s instructions. DNA integrity was examined by 1% agarose gel electrophoresis, and DNA purity and concentration were measured using NanoDrop and Qubit (Thermo Fisher Scientific, Waltham, MA, USA). The resulting eDNA solutions were quickly stored at −20 °C until PCR amplification. PCR amplification targeted the mitochondrial 12S rRNA gene region using the MiFish-U/E primer set (F: 5′-GTYGGTAAAWCTCGTGCCAGC-3′, R: 5′-CATAGTGGGGTATCTAATCCYAGTTTG-3′) [24]. The PCR reaction mixture (20 μL) included: 2 μL template DNA (10 ng/μL), 0.8 μL each of forward and reverse primers (10 μmol/L), 2 μL dNTPs, 4 μL 5× FastPfu buffer, 0.4 μL FastPfu polymerase (TransGen Biotech, Beijing, China), and 10 μL ddH_2_O. The PCR program was: 95 °C for 5 min initial denaturation; 27 cycles of 95 °C for 30 s denaturation, 55 °C for 30 s annealing, 72 °C for 45 s extension; and a final extension at 72 °C for 10 min. In each PCR run, we used ddH_2_O as template for a PCR negative control. Each sample was amplified in triplicate. To monitor potential contamination during the amplification process, a negative control (ddH_2_O template) was included in each PCR plate. The triplicate PCR products for each sample were pooled and examined on 2% agarose gels; no target bands were observed in any of the negative controls. After confirmation, the pooled replicate PCR products were purified using AMPure XP magnetic beads (Beckman Coulter, Brea, CA, USA) at a 1:1 bead-to-sample volume ratio (50 μL beads per 50 μL PCR product) to remove primer dimers and non-target fragments.

Purified amplicons were quantified using a Qubit dsDNA HS Assay Kit (Thermo Fisher Scientific, Waltham, MA, USA). Sequencing libraries were then constructed using the NEBNext Ultra II DNA Library Prep Kit for Illumina (New England Biolabs, Ipswich, MA, USA) with dual-index barcodes. The resulting indexed libraries were purified and size-selected using 0.8× AMPure XP beads. Library quality control was subsequently performed to assess concentration and fragment size distribution using a Qubit fluorometer (Thermo Fisher Scientific, Waltham, MA, USA) and a Qsep400 system (Bioptic, Taiwan, China), respectively. Based on these quality control measurements, the individual libraries were pooled at equimolar concentrations. The final pooled library exhibited an average insert size of 349 bp and a concentration of 42.76 ng/μL. Sequencing was performed on an Illumina NovaSeq 6000 platform (San Diego, CA, USA) using the NovaSeq 6000 SP Reagent Kit v1.5 with a paired-end 150 bp (PE150) sequencing strategy.

### 2.3. Sequence Processing

We performed quality assessment and trimming of raw sequence data using FastQC (v0.11.9) combined with SolexaQA++ (v3.1.7.2) (Phred < 10) [18]. Paired-end sequences were merged using FLASH (v1.2.11), and then length anomalies (theoretical length 229 ± 25 bp) and primer sequences (allowing 3 bp mismatches) were sequentially removed using SeqKit (v2.13.0) and Tag Cleaner (0.16). Denoising and chimera removal were performed using USEARCH v11. Specifically, all filtered and merged sequences were first pooled and dereplicated using the fastx_uniques algorithm, with low-abundance sequences discarded by setting the minimum abundance threshold to 4 (using parameter -minuniquesize 4). Subsequently, de novo chimera detection and removal were performed on the unique sequences using the uchime3_denovo algorithm. Finally, the non-chimera unique sequences were denoised using the unoise3 algorithm to generate zero-radius operational taxonomic units (zOTUs). Individual sample abundance tables were then mapped against these representative sequences using the usearch_global algorithm [25]. Finally, species annotation was performed by BLASTn (2.16.0+) against the MiFish (MitoFish) database (http://mitofish.aori.u-tokyo.ac.jp/, accessed on 16 July 2026) and NCBI databases [26]. zOTUs were first compared to the MiFish database; those without clear matches were further compared to the NCBI database. Species assignment thresholds were set at ≥98% similarity and ≥95% coverage. When multiple species matched equally or the database did not provide species-level information, we retained only genus- or family-level assignments. To avoid false positives, we included blank filter negative controls.

### 2.4. Data Processing

To quantify short-timescale variation in fish communities at each sampling station, we used β-diversity analysis based on the Bray–Curtis distance. First, we converted absolute species abundance data to relative abundances to remove bias due to differences in sequencing depth. To compare the mean temporal change rates (Bray–Curtis dissimilarities) among stations, we performed a one-way analysis of variance (ANOVA) followed by Tukey’s honest significant difference (HSD) post-hoc test for pairwise comparisons. Normality of the data was assessed using the Shapiro–Wilk test, and homogeneity of variances was assessed using Levene’s test. We performed similarity percentage analysis (SIMPER) to identify the contribution of individual families to temporal community dissimilarity. Species diversity was characterized using three standard indices: Margalef richness index [27], Shannon–Wiener diversity index [28], and Pielou evenness index [29]. All statistical analyses were performed in R (version 4.5.2) using the vegan package (version 2.7–3) for community ecology analyses and the stats package (v4.5.0) for ANOVA and post-hoc tests. Visualization was performed with ggplot2 package (v4.0.3 and Origin 2026. SPSS 20 was used for auxiliary statistical tests, and ArcGIS desktop 10.8 for spatial data visualization.

## 3. Results

### 3.1. Species Composition

Using eDNA metabarcoding, we obtained 5,824,043 raw sequences. After quality filtering, denoising, and chimera removal, we retained 341 fish-assigned zOTUs. These zOTUs were resolved and collapsed into 254 unique fish species belonging to 174 genera, 82 families, 23 orders, and 2 classes (Table S1). Among these, the class Actinopterygii contributed 249 species, representing 20 orders, with Perciformes being the most dominant order (162 species, 63.78% of total species). This was followed by Clupeiformes (28 species), Anguilliformes (12 species), Scorpaeniformes (11 species), Pleuronectiformes (6 species), Tetraodontiformes (6 species), and Mugiliformes (6 species). In addition, Gadiformes, Lophiiformes, Elopiformes, Aulopiformes, Gasterosteiformes, Myctophiformes, Synbranchiformes, Beryciformes, Cypriniformes, Siluriformes, Gonorhynchiformes, Atheriniformes, and Ophidiiformes each had fewer than five species. The class Chondrichthyes contributed five species from three orders: Myliobatiformes (3 species), Orectolobiformes (1 species), and Rajiformes (1 species).

**Figure 1:**
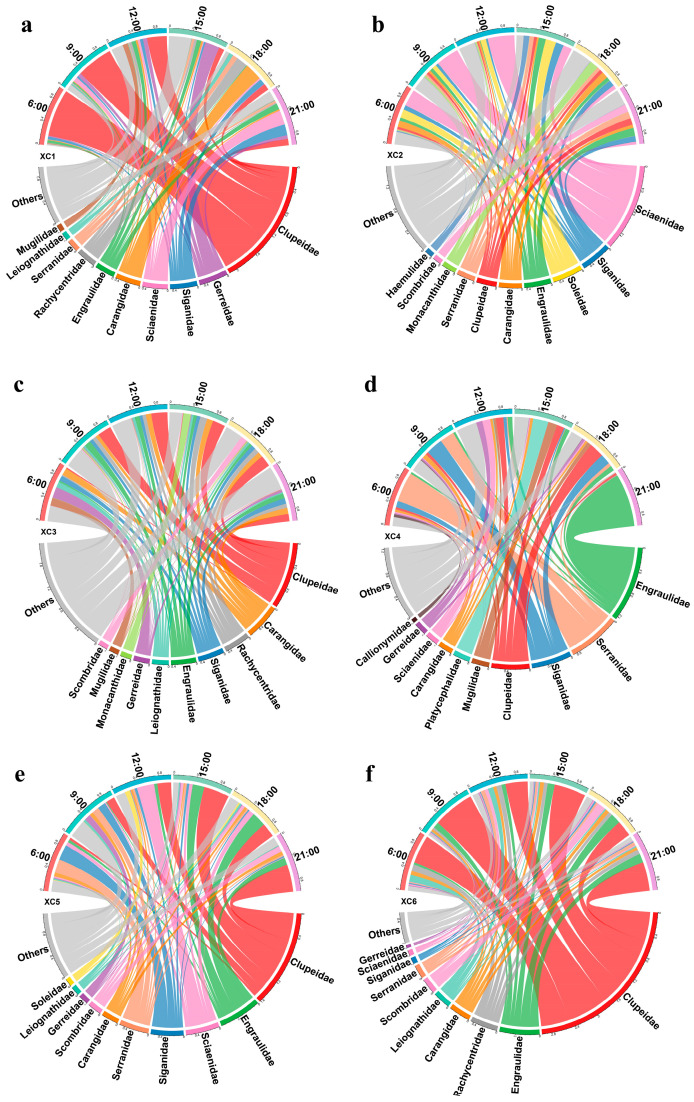
Fish species composition (family level) at different times for each sampling station. (**a**–**f**). represent fish species composition (family level) at stations XC1, XC2, XC3, XC4, XC5, and XC6, respectively, at different time points.

In terms of family-level relative abundance across stations, Clupeidae was the most dominant taxon, reaching a relative abundance of 82.87% at the lagoon edge (XC1, R1) and also dominating at the offshore 5 m isobath (XC5) and 10 m isobath (XC6) (48.04% and 50.73%, respectively, at R6). Engraulidae showed an extreme peak (93.12%) at the narrowest point of the lagoon inlet (XC4) during the R6 period, representing the most striking dynamic feature of that station. Additional families among the top 10 in relative abundance across stations included Leiognathidae, Monacanthidae, Rachycentridae, Siganidae, Soleidae, Serranidae, Scombridae, Carangidae, Haemulidae, Sciaenidae, Callionymidae, Gerreidae, Platycephalidae, and Mugilidae (Table S2, Figure 1).

### 3.2. Community Difference Analysis

**Figure 2:**
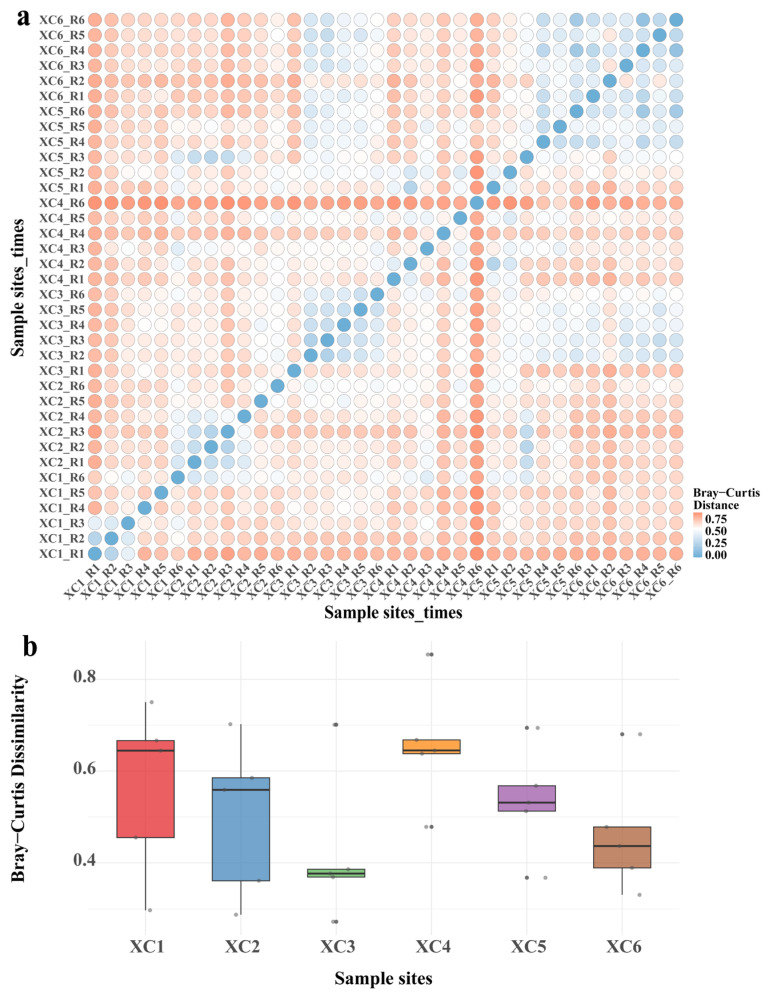
Bray–Curtis distance matrix among samples and temporal change rates at each sampling station. (**a**). Bray–Curtis distance matrix among all samples; (**b**). Distribution of temporal change rates at each sampling station.

To quantify community differences among time points within each station, we calculated Bray–Curtis dissimilarities between consecutive time points for each station (Figure 2a). The lagoon inlet (XC4) had the highest mean community change rate (0.657 ± 0.134), significantly higher than all other stations (*p* < 0.05). The change rate at XC4 peaked during the R5–R6 period (18:00–21:00) at 0.854, the highest value among all station–time-period combinations, indicating that the evening period was the window of most intense change in fish community structure at the lagoon inlet. In contrast, the mangrove creek area (XC3) had the lowest mean change rate (0.421 ± 0.163), reflecting the relative stability of the community structure at this site on a diurnal scale.

Notably, the lagoon edge (XC1) had a mean change rate (0.563 ± 0.183) second only to XC4 and higher than those of XC5 (0.535 ± 0.117) and XC6 (0.463 ± 0.133) (Table S3, Figure 2b).

### 3.3. Station-Level Diversity and Temporal Fluctuation

**Figure 3:**
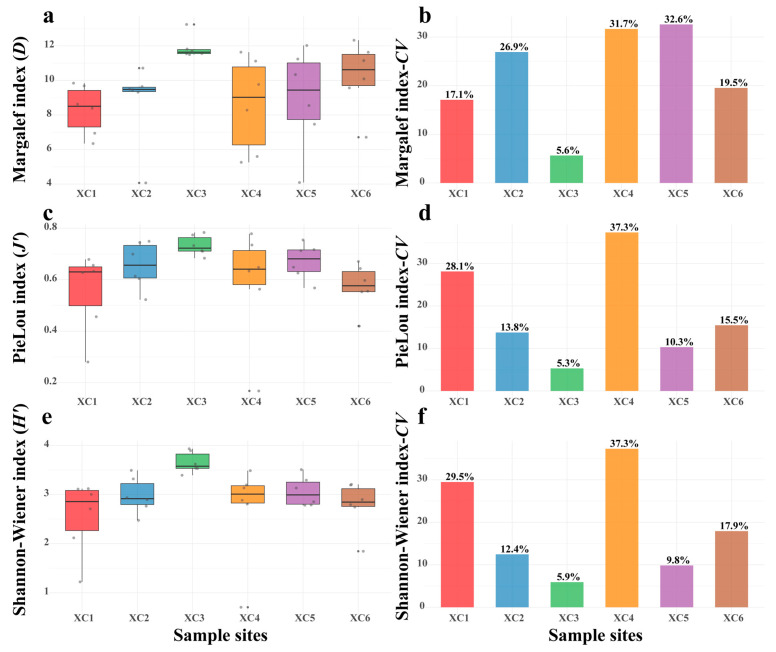
Biodiversity indices and their coefficients of variation across sampling stations (XC1–XC6). Panels (**a**,**c**,**e**) show the distribution of three diversity indices at each station across all time points. (**a**) Margalef richness index (*D*), (**c**) Pielou evenness index (*J′*), and (**e**) Shannon–Wiener diversity index (*H′*). Panels (**b**,**d**,**f**) show the corresponding coefficients of variation (*CV* = standard deviation/mean × 100%) for each station, indicating the degree of temporal fluctuation across the six sampling time points (R1–R6). Higher CV values indicate greater temporal instability. (**b**) Coefficient of variation of Margalef richness index, (**d**) Coefficient of variation in Pielou evenness index, and (**f**) Coefficient of variation in Shannon–Wiener diversity index.

The mangrove creek area (XC3) had the highest and most stable diversity across all indices. In contrast, the lagoon inlet (XC4) showed the greatest temporal fluctuations, with the coefficient of variation in the Shannon index substantially higher than that of XC3. Lagoon interior stations (XC1, XC2) showed intermediate diversity levels, with XC1 exhibiting greater temporal instability than XC2. Offshore stations (XC5, XC6) showed diversity levels between those of the lagoon interior and the mangrove area, with XC5 slightly higher than XC6 (Figure 3).

### 3.4. Key Species

**Figure 4:**
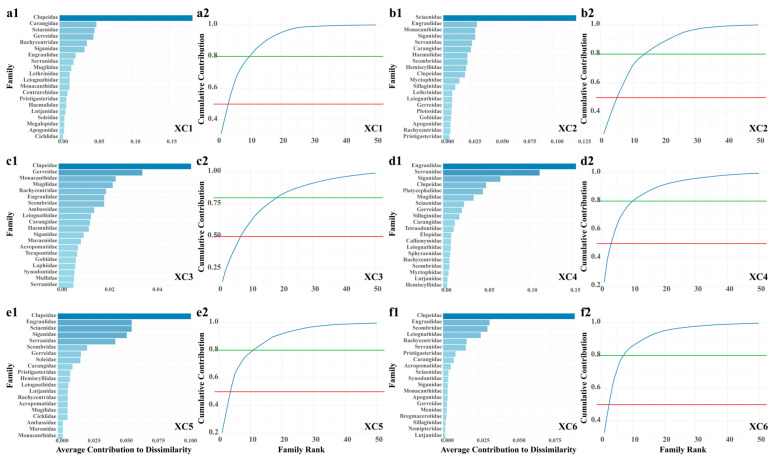
Top 20 families by mean contribution to dissimilarity and cumulative contribution percentages at different sampling stations. (**a1**–**f1**). Top 20 families by mean contribution to dissimilarity at stations XC1, XC2, XC3, XC4, XC5, and XC6, respectively. (**a2**–**f2**). Cumulative contribution percentages at stations XC1, XC2, XC3, XC4, XC5, and XC6, respectively. Red line indicates 50% cumulative contribution, green line indicates 80% cumulative contribution.

SIMPER analysis identified the families contributing most to temporal community dissimilarity at each station (Table S4, Figure 4). At the lagoon inlet (XC4), Engraulidae had the highest mean contribution (15.46%), followed by Serranidae (11.23%) and Siganidae (6.66%). The high contribution of Engraulidae at XC4 was mainly due to its extreme abundance peak during the R6 period, which is highly consistent with the results of the community difference analysis and diversity analysis. The cumulative contribution curve showed that the top three families explained approximately 50% of the total variation, and the top ten families explained approximately 80% of the total variation, indicating that temporal community dissimilarity was mainly driven by a few dominant families (Figure 4(d1,d2). Within the lagoon interior (XC1), Clupeidae had the highest contribution (18.32%), reflecting its consistently high abundance and dominant role in community structure. The cumulative contribution curve also indicated that temporal community dissimilarity was mainly driven by a few dominant families (Figure 4(a1,a2)). At the mangrove creek area (XC3), Clupeidae also contributed the most (5.56%), but the cumulative contribution curve showed that the top ten families explained only about 61% of the total variation, indicating that community changes in this region are distributed among more families (Figure 4(c1,c2)). At offshore stations (XC5, XC6), Clupeidae and Engraulidae were the two families with the highest contributions, generally consistent with the pattern at the lagoon inlet (Figure 4(e1,e2,f1,f2)).

### 3.5. Diel Variation in Clupeidae eDNA Signals

**Figure 5:**
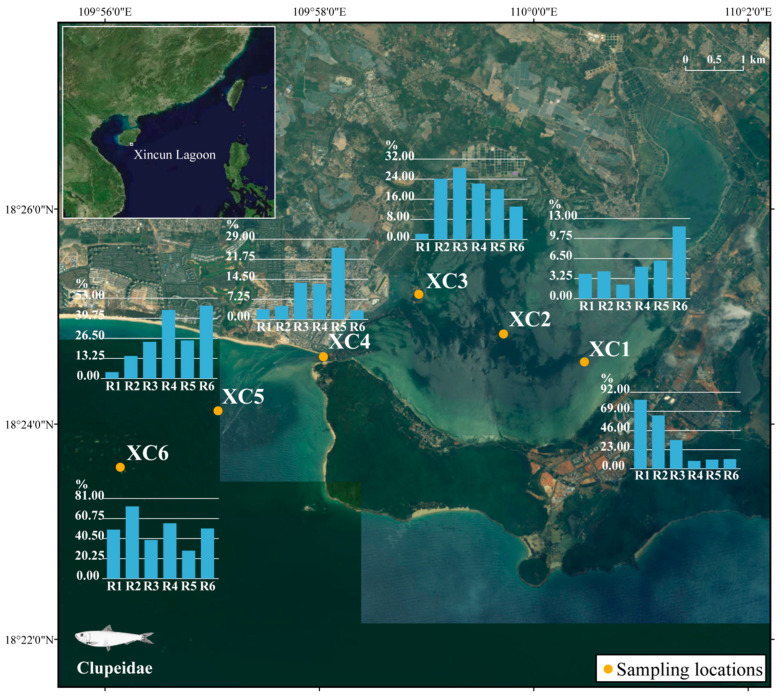
Spatiotemporal distribution of Clupeidae. Note: blue bars indicate relative abundance of Clupeidae at different time points for each sampling station.

Based on the relative abundance distribution and dissimilarity contribution of Clupeidae across stations and time points, we further analyzed the diurnal migration pattern of this family (Table S5, Figure 5). Clupeidae showed a notable spatiotemporal pattern in eDNA signals: inside the lagoon (XC1), Clupeidae relative read abundance peaked during the R1 period (6:00) at 82.87% and then declined; at the lagoon inlet (XC4), Clupeidae abundance was low but showed a small peak during the R5 period (18:00) at 25.92%; in offshore waters (XC5, XC6), Clupeidae abundance increased during the R5–R6 periods (18:00–21:00), reaching 25.09–48.04% and 28.28–50.73%, respectively. This temporal sequence is consistent with the hypothesis that Clupeidae eDNA signals may reflect movement from the lagoon interior, through the inlet, to offshore waters during the evening. The high change rate at XC4 during the R5-R6 period (0.854) and the synchronous high relative signal of Engraulidae are consistent with the interpretation that the lagoon inlet represents a zone of pronounced diel change in eDNA-based fish community signals, suggesting a possible passageway role.

## 4. Discussion

### 4.1. Spatial Functional Zonation of the Lagoon Ecosystem

A key finding of this study is the spatial differentiation of fish community diversity and temporal dynamics along the lagoon–inlet–open sea gradient, suggesting eDNA-based diel connectivity signals along the gradient.

The lagoon inlet (XC4) showed the most pronounced temporal dynamics in eDNA-based community composition. These patterns were likely driven by a few taxa, such as Engraulidae. This finding is consistent with global observations of diurnal activity rhythms of clupeiform fishes [30,31]. Castillo-Rivera and Kobelkowsky [31] documented distinct activity peaks at dusk for two sympatric *Brevoortia* species in a Mexican coastal lagoon. Their distribution patterns were closely linked to light intensity and feeding activity. Joyeux [30] demonstrated that juvenile *Brevoortia tyrannus* enter estuarine systems during dusk flood tides, providing a mechanistic explanation for the evening pulse we observed at the Xincun Lagoon inlet. The ecological characteristics of Clupeidae and Engraulidae, including schooling behavior, filter-feeding mode, and sensitivity to light gradients, may explain why these taxa appear to dominate the dusk pulse [32,33]. The characterization of the lagoon inlet as an important ecological corridor in coastal lagoons is also supported by hydrodynamic modeling studies. Ghezzo, De Pascalis [11] used Lagrangian particle tracking simulations to show that larval exchange probabilities between coastal lagoons and adjacent seas are generally low. However, the physical morphology of the channel profoundly influences the strength and direction of connectivity. For Xincun Lagoon, the single and narrow tidal inlet, approximately 150 m wide, constitutes a hydrodynamic bottleneck where flow velocities are highest. This creates a physical constriction that concentrates biological passage in a spatially restricted area [11,34]. Thus, the extreme temporal variability we observed in eDNA signals at XC4 aligns with the physical expectation of the tidal inlet as a key point of ecological connectivity. Similar functions have been documented in other lagoon systems. Widening and deepening of the El Estacio channel in the Mar Menor lagoon in Spain led to major changes in fish assemblage composition, including the immigration of new species and the decline of traditional fisheries that depended on the channel for fish migration [3]. That study highlighted the ecological importance of tidal inlets and the potential risk that physical alterations could substantially disrupt connectivity processes.

The mangrove creek area (XC3) maintained high and stable eDNA-based diversity. This finding is consistent with the established role of mangrove habitats as biodiversity hotspots and key nursery grounds in tropical coastal seascapes [35,36]. The complex structure provided by mangrove pneumatophores, combined with moderate hydrodynamic mixing in the creek environment, likely creates favorable conditions that support both high species richness and stable community structure [37]. Dorenbosch, Verberk [38] similarly reported that mangrove habitats in the Indo-Pacific region provide important juvenile habitat for multiple coral reef-associated fish species. The spatial arrangement of mangroves relative to other habitats significantly influences fish assemblage composition on adjacent reefs. In the Xincun Lagoon system, the mangrove creek area appears to represent an ecotone where transitional waters combine with structurally complex habitats to maintain community stability throughout the diurnal cycle. This interpretation is supported by the finding that community variation at XC3 was distributed among a larger number of families [39]. From a functional perspective, the mangrove creek serves both as a biodiversity hotspot and as a buffer against temporal fluctuations [37,40].

In contrast to the high diversity and stability at the mangrove creek, the lagoon interior stations exhibited intermediate diversity levels with markedly higher temporal variability at the lagoon edge (XC1) than at the center (XC2). This pattern aligns with the ‘confinement’ theory of Pérez-Ruzafa and Marcos [41], which posits that isolation from marine influence is a primary factor shaping biological assemblages in coastal lagoons [3]. Our results also suggest heterogeneity within the lagoon interior. The central area (XC2) exhibited greater temporal stability than the lagoon edge (XC1), possibly due to buffering from marine external influences and local edge effects. The elevated variability at XC1 may reflect multiple interacting mechanisms. First, endogenous diel rhythms of lagoon-resident fishes may produce community composition changes unrelated to cross-boundary connectivity, including crepuscular foraging movements [22]. Second, temperature-driven vertical migration in shallow, stratified waters at the lagoon edge could alter the detectable eDNA community at the sampling depth [42,43]. Third, diel variation in predation risk may shape the temporal pattern of prey fish activity in exposed shallow habitats [23]. The interaction of these processes with restricted hydrodynamic connectivity likely produces the intermediate but temporally dynamic patterns observed at the lagoon interior [3].

The offshore stations, located at the 5 m and 10 m isobaths, exhibited marked differences in eDNA-based diversity and temporal variability despite their proximity to the lagoon inlet. The nearshore area (XC5) experienced greater diurnal change in eDNA community signals than the more distant open waters (XC6). This suggests that the ecological influence of the lagoon, as reflected by eDNA signals, extends detectably to the 5 m isobath but is substantially reduced at the 10 m isobath [44]. This spatial pattern may reflect a hydrodynamic phenomenon. The water mass exiting the lagoon through the narrow tidal inlet carries entrained biological material into the adjacent nearshore area. As distance from the inlet mouth increases, this lagoon-influenced water mass gradually mixes with the surrounding open-sea water mass. This creates a decreasing gradient of lagoon-derived biological influence [45,46]. At XC5, located directly outside the lagoon inlet, the fish community retains a detectable signal of lagoon connectivity. At XC6, farther from the inlet and with greater water depth, the lagoon signal weakens and the open-sea water mass gradually dominates. This pattern is consistent with the threshold distance framework established in ecological studies of tropical nearshore ecosystems. Berkström, Eggertsen [45] demonstrated that the influence of mangrove and seagrass nursery habitats on coral reef fish community structure drops sharply beyond a threshold of approximately 8 km from nursery areas. Some nursery-associated species were completely absent on reefs 80 km from mangroves and 12 km from seagrass beds. Although the spatial scale of the Xincun Lagoon system is considerably smaller than the expansive Bazaruto Archipelago seascape studied by [45], the underlying ecological principle is applicable across diverse nearshore environments. The biological influence of a habitat decays nonlinearly with distance, and identifiable threshold distances exist beyond which that influence becomes negligible. Furthermore, several authors have emphasized the importance of the physical matrix separating habitat patches, including water depth and substrate type, as a moderator of connectivity strength [45,46]. In the Xincun Lagoon system, the transition from shallow lagoon-influenced waters at the 5 m isobath to deeper, more pelagic-dominated waters at the 10 m isobath likely represents such a matrix-mediated transition.

### 4.2. Temporal Dynamics: Diurnal Migration and Connectivity

Diurnal-scale community reorganization was most pronounced at the lagoon inlet (XC4) and the innermost edge station (XC1). At XC4, the evening peak in eDNA signals is consistent with patterns that could reflect passage through the inlet. At XC1, the elevated variability likely reflects endogenous diel rhythms of lagoon-resident fishes, such as crepuscular foraging movements and temperature-driven vertical migration occurring in shallow, thermoclined lagoon edge waters [22,42,43].

The spatiotemporal distribution of Clupeidae along the lagoon–inlet–open sea gradient is consistent with a directed, diurnal-scale movement pattern. Clupeidae eDNA signals were strongest inside the lagoon during the day. They were detected at the inlet in the evening and in offshore waters at night. The synchronous high relative signal of Engraulidae at XC4 during the evening further suggests that the tidal inlet may serve as a passage corridor for multiple small pelagic schooling fishes [47,48]. The consistent importance of Clupeidae across all stations along the environmental gradient highlights its potential role as a major participant in diurnal connectivity throughout the lagoon [39]. According to tidal data for Xincun Bay on 24 January 2024 (Figure S1), low tide occurred at approximately 6:00, and flood tide occurred in the evening. Thus, the observed evening increase in Clupeidae and Engraulidae eDNA signals at the inlet (XC4) coincided with the flood tide, which may have facilitated fish passage through the narrow channel. However, eDNA data alone cannot distinguish between active swimming and passive transport by tidal currents. The observed pattern could reflect selective tidal stream transport, a behavior documented in clupeiform fishes whereby fish use tidal currents to assist movement between habitats [49]. Alternatively, the pattern could reflect passive advection of eDNA from upstream areas. These alternative explanations cannot be resolved with eDNA data alone and should be investigated in future studies using complementary approaches such as acoustic telemetry or net-based sampling during tidal cycles.

The schooling behavior and strong sensitivity to light of clupeiform fishes may explain why they appear to be dominant participants in the diurnal eDNA connectivity signals observed in this lagoon system [32,33,49,50]. Multiple ecological drivers may contribute to the observed diurnal pattern in eDNA signals. Flood tide in the evening could potentially facilitate fish movement [49]. Second, the timing of migration during twilight is consistent with the “antipredation window” hypothesis of Clark and Levy [51]. This hypothesis posits that the brief window of moderate light at dawn and dusk allows prey fish to move between habitats with reduced risk of detection by visual predators. Danilowicz and Sale [52] noted that predation intensity on *Haemulon flavolineatum* was significantly lower at night and during twilight than during the day. Rooker, Dance [23] observed that the fastest movement rates of prey fish crossing a high-risk channel occurred during twilight. Third, the timing of movement may be linked to the diel vertical migration of zooplankton prey. Zooplankton typically ascend to surface waters at night, providing feeding opportunities for planktivorous clupeiform fishes [42,43,53]. The combined effects of tidal assistance, minimization of predation risk, and prey availability may together shape the timing of the observed patterns.

### 4.3. Contributions and Limitations of the eDNA Method in Diurnal Connectivity Studies

This study demonstrates the utility of eDNA metabarcoding for capturing fine-scale diel variation in fish community composition and distribution. This information is difficult to obtain with traditional sampling methods. The non-invasive nature of eDNA sampling, its high-throughput taxonomic identification capacity, and its sensitivity to transiently appearing species were essential to our analysis [12,13]. In the Xincun Lagoon system, the concentrated signals of Clupeidae and Engraulidae at the narrow tidal inlet during the evening represent transient phenomena within a limited spatiotemporal window. Obtaining similar resolution with fishing nets would be challenging due to gear selectivity, target avoidance, and the inability to deploy synchronously at multiple stations and time points [54]. Furthermore, traditional capture-based surveys in narrow channels would cause habitat disturbance, which is particularly problematic in sensitive lagoon ecosystems. Thus, eDNA aligns with the global trend toward non-destructive monitoring [16,55].

However, several limitations of the eDNA approach warrant consideration. First, eDNA persistence varies with environmental conditions such as temperature, UV radiation, pH, and microbial activity [14,56]. In warm tropical lagoons, degradation rates are expected to be faster, which may enhance detection of recent biological activity [57,58]. However, we did not measure degradation kinetics, so some temporal blurring cannot be ruled out. Second, eDNA does not provide information on the demographic attributes, such as body length, age stage, or absolute abundance. This information is essential for distinguishing juvenile from adult movement [59]. The inferred movement pattern represents a population- or community-level signal. Third, eDNA does not distinguish between DNA released by living fish, carcasses, predator-released DNA, or mucus from sedentary individuals [60]. The high relative eDNA signal of Engraulidae at XC4 at 21:00 could partially result from predation events or advected DNA rather than the presence of live fish at that location. Although the temporal coherence of the observed pattern and the known behavioral ecology of Engraulidae are consistent with active movement, alternative sources cannot be ruled out. Fourth, eDNA data directly show fine-scale diel variation in community composition and relative eDNA signal distribution across the lagoon–inlet–offshore gradient. Interpretations of these patterns as evidence of active fish migration, directed movement pathways, or corridor function are inferences. These interpretations represent our best working hypotheses, consistent with the observed data and established ecological knowledge, but they are not direct observations of individual fish movement. Fifth, our sampling was conducted on a single day in January, during the dry season and low tide slack period. Diurnal patterns may differ during the wet season, during spring tides, or under different environmental conditions. Seasonal and tidal-cycle effects therefore require further investigation. The results presented here should be interpreted as representative of dry-season, low-tide conditions in Xincun Lagoon.

Despite these limitations, the convergence of multiple lines of evidence provides support for our main findings. These include the temporal coherence of community turnover, the synchronous timing of high relative signals in multiple taxa, consistency with behavioral ecology theories, and rigorous sampling design. The ecological interpretations—that these patterns may reflect movement through the inlet and corridor function—represent our best working hypotheses. Future studies could strengthen inference by integrating eDNA with acoustic telemetry to validate movement pathways [23]; using multi-primer approaches to broaden taxonomic coverage [61]; conducting local DNA barcoding to enrich reference databases [18]; and performing controlled degradation experiments to better constrain temporal resolution.

## 5. Conclusions

In this study, we used eDNA metabarcoding with diurnal-scale repeated sampling to investigate spatiotemporal dynamics of fish community connectivity in Xincun Lagoon. Our findings suggest three main patterns. First, fish community structure shows spatial functional differentiation along the lagoon–inlet–open sea gradient. The mangrove creek maintained the highest and most stable diversity, functioning as a biodiversity hotspot. The lagoon edge exhibited greater temporal dynamics than the central area. Offshore stations showed intermediate diversity, with lagoon influence extending to the 5 m isobath but weakening at the 10 m isobath. Second, the narrow tidal inlet was the region with the greatest temporal change rate in community composition. This suggests a potential corridor role for diurnal fish movement. Third, the spatiotemporal distribution of Clupeidae is consistent with a directed movement pattern. eDNA signals were strongest inside the lagoon during the day, at the inlet in the evening, and offshore at night. This pattern is consistent with established behavioral ecological frameworks. Our results demonstrate how eDNA can capture fine-scale diel variation in fish distribution that is difficult to obtain through conventional surveys. They also provide practical evidence for the conservation and management of these sensitive transitional waters.

## Figures and Tables

**Table 1:** Characteristics of the six sampling stations along the lagoon–inlet–offshore gradient.

Station	Location	Hydrodynamic Characteristics	Habitat Type
XC1	Lagoon interior edge, farthest from inlet	Weakest water exchange, most enclosed	Still-water, enclosed lagoon
XC2	Lagoon center	Limited tidal influence, greater depth	Typical lagoon interior
XC3	Mangrove creek within lagoon	Periodic tidal inundation, moderate mixing	Transitional mangrove habitat
XC4	Narrowest point of lagoon inlet	Strongest hydrodynamic conditions	Core tidal inlet
XC5	Offshore 5 m isobath	Influenced by lagoon outflow and open-sea tides	Nearshore transitional
XC6	Offshore 10 m isobath	Relatively stable, minimal terrestrial influence	Open marine

## Data Availability

Data available on request from the authors.

## Supplementary Materials

The following supporting information can be downloaded at: https://www.mdpi.com/article/10.3390/ani16142268/s1, Table S1: Stepwise summary of reads, zOTUs, and taxa retained through the bioinformatics filtering pipeline. Table S2: Fish species composition (family level) at different times for each sampling station. Table S3: Distribution of temporal change rates at each sampling station. Table S4: Top 20 families by mean contribution to dissimilarity and cumulative contribution percentages at different sampling stations. Table S5: Spatiotemporal distribution of Clupeidae (%). Figure S1. Tidal curve of Xincun Bay on 24 January 2024.
