# Supplementary material for: eDNA Metabarcoding Reveals Diel Connectivity Dynamics of Fish Communities in Xincun Lagoon, Hainan
Source: Animals (Basel). 2026 Jul 22;16(14):2268. doi: 10.3390/ani16142268 (PMC13403707; doi:10.3390/ani16142268)
Supplement: Supplementary file 1 [file animals-16-02268-s001.zip › Table S.pdf]

Table S1 Stepwise summary of reads, zOTUs, and taxa retained through the bioinformatics filtering pipeline.

Pipeline stage	Count	Description
Unique tags	70,776	All non-redundant sequences obtained after initial dereplication of raw reads.
Filtered unique tags	42,063	Unique sequences retained after abundance filtering using the -minuniquesize 4 threshold.
zOTUs	1,014	Amplicon sequence variants generated via the unoise3 denoising pipeline.
Fish-assigned zOTUs	341	zOTUs successfully matched and taxonomically assigned to fish taxa.
Unique taxa	274	Non-redundant taxonomic units calculated by collapsing intraspecific zOTUs while retaining unresolved genus/family-level sequences.
Identified species	254	Unique taxonomic units strictly resolved and validated at the binomial species level.

Table S2 Fish species composition (family level) at different times for each sampling station.

Sites	Family	R1	R2	R3	R4	R5	R6
XC1	Clupeidae	82.87	64.04	34.59	9.40	10.99	11.57
	Gerreidae	1.85	6.28	7.37	25.68	1.07	6.23
	Siganidae	4.68	3.07	5.49	2.99	10.08	19.20
	Sciaenidae	0.49	8.10	4.06	1.62	3.03	25.18
	Carangidae	1.32	1.20	3.43	3.12	30.20	2.67
	Engraulidae	4.21	2.22	7.59	7.86	1.22	10.31
	Rachycentridae	0.40	1.28	4.97	4.48	20.25	1.97
	Serranidae	0.31	0.70	1.17	4.36	8.98	4.27
	Leiognathidae	0.32	2.82	1.11	0.74	7.28	0.86
	Mugilidae	1.13	0.14	9.25	0.25	0.34	0.49
	Others	2.41	10.15	20.98	39.51	6.55	17.25
XC2	Sciaenidae	29.52	40.54	46.98	15.68	6.85	4.59
	Siganidae	7.84	5.13	3.23	14.13	11.17	10.63
	Soleidae	14.77	7.62	9.74	18.59	0.38	0.29
	Engraulidae	5.45	5.70	1.22	12.43	6.70	13.62
	Carangidae	11.46	3.81	2.91	3.15	11.37	5.83
	Clupeidae	4.02	4.42	2.25	5.17	6.16	11.70
	Serranidae	1.09	3.68	0.93	7.30	6.06	11.70
	Monacanthidae	1.51	3.18	2.69	0.53	15.87	0.34
	Scombridae	0.25	0.25	0.30	0.38	0.27	13.34
	Haemulidae	1.90	0.34	0.52	10.13	0.45	0.34
	Others	22.18	25.34	29.23	12.50	34.73	27.63
XC3	Clupeidae	2.24	24.20	28.45	22.22	20.00	13.05
	Carangidae	12.55	6.02	9.61	10.97	9.17	10.85
	Rachycentridae	2.20	7.11	6.14	12.46	8.83	7.33
	Siganidae	9.24	6.29	5.39	6.06	7.47	9.54
	Engraulidae	2.64	11.22	8.37	5.71	5.13	9.07
	Leiognathidae	9.83	3.58	4.57	3.85	3.94	2.31
	Gerreidae	19.44	1.09	0.77	1.17	0.51	4.28
	Monacanthidae	0.35	2.26	0.71	13.20	2.07	0.27
	Mugilidae	13.54	0.49	0.28	0.99	0.47	0.69
	Scombridae	0.49	1.56	0.78	1.15	11.44	0.93
	Others	27.47	36.18	34.93	22.21	30.96	41.67
XC4	Engraulidae	3.40	3.97	8.33	7.70	8.78	93.12
	Serranidae	52.44	25.67	1.85	0.78	5.99	0.87
	Siganidae	10.96	29.76	5.67	2.32	18.34	0.45
	Clupeidae	3.67	4.85	13.18	12.84	25.92	3.28
	Mugilidae	0.42	0.38	4.59	16.48	7.57	0.29
	Platycephalidae	0.05	0.07	1.19	27.08	0.27	0.00
	Carangidae	3.95	7.69	4.32	4.77	3.70	0.18

	Sciaenidae	2.44	1.34	14.04	1.52	2.25	0.23
	Gerreidae	2.26	3.22	10.64	0.63	4.55	0.07
	Callionymidae	4.84	1.12	0.12	0.17	0.44	0.02
	Others	15.55	21.94	36.06	25.71	22.18	1.48
XC5	Clupeidae	4.20	14.89	24.20	45.16	25.09	48.04
	Engraulidae	4.62	1.66	5.71	20.80	25.06	12.71
	Sciaenidae	6.68	2.18	30.58	5.95	9.98	0.91
	Siganidae	26.96	14.41	4.64	4.26	2.35	2.06
	Serranidae	23.76	12.60	3.41	3.93	3.02	2.75
	Carangidae	6.63	6.19	3.44	1.70	4.62	6.07
	Scombridae	2.38	0.37	0.25	4.69	5.37	10.46
	Gerreidae	1.40	10.93	0.84	1.53	1.34	0.52
	Leiognathidae	2.29	4.35	0.76	2.35	3.71	1.48
	Soleidae	0.91	0.33	8.98	0.00	2.61	0.17
	Others	20.18	32.07	17.18	9.62	16.87	14.82
XC6	Clupeidae	49.59	73.00	39.12	55.99	28.28	50.73
	Engraulidae	3.72	10.42	7.06	12.48	17.77	15.39
	Rachycentridae	11.58	3.73	9.41	6.55	6.55	8.09
	Carangidae	6.93	4.14	5.48	6.81	7.38	5.32
	Leiognathidae	12.12	0.80	9.64	2.52	2.43	2.26
	Scombridae	0.64	0.07	6.12	1.90	15.32	1.73
	Serranidae	1.53	0.44	7.48	3.81	3.72	5.65
	Siganidae	1.81	1.22	1.52	1.81	2.97	1.64
	Sciaenidae	2.12	1.63	1.48	0.98	2.48	0.93
	Gerreidae	1.16	0.35	1.51	0.53	1.05	0.45
	Others	8.80	4.20	11.18	6.62	12.05	7.81

Table S3 Distribution of temporal change rates at each sampling station.

Site	Time_Pair	Bray–Curtis Dissimilarity
XC1	R1→R2	0.297
XC1	R2→R3	0.455
XC1	R3→R4	0.644
XC1	R4→R5	0.750
XC1	R5→R6	0.666
XC2	R1→R2	0.361
XC2	R2→R3	0.288
XC2	R3→R4	0.559
XC2	R4→R5	0.702
XC2	R5→R6	0.585
XC3	R1→R2	0.701
XC3	R2→R3	0.272
XC3	R3→R4	0.369
XC3	R4→R5	0.377
XC3	R5→R6	0.386
XC4	R1→R2	0.478
XC4	R2→R3	0.645
XC4	R3→R4	0.668
XC4	R4→R5	0.638
XC4	R5→R6	0.854
XC5	R1→R2	0.513
XC5	R2→R3	0.694
XC5	R3→R4	0.568
XC5	R4→R5	0.368
XC5	R5→R6	0.531
XC6	R1→R2	0.478
XC6	R2→R3	0.680
XC6	R3→R4	0.437
XC6	R4→R5	0.389
XC6	R5→R6	0.331

Table S4 Top 20 families by mean contribution to dissimilarity and cumulative contribution percentages at different sampling stations.

Sites	Rank	Family	Avg_Contribution	Cumulative_Contribution
XC1	1	Clupeidae	0.1832	0.3115
	2	Carangidae	0.0506	0.3976
	3	Sciaenidae	0.0480	0.4792
	4	Gerreidae	0.0466	0.5584
	5	Rachycentridae	0.0376	0.6223
	6	Siganidae	0.0343	0.6807
	7	Engraulidae	0.0219	0.7180
	8	Serranidae	0.0191	0.7505
	9	Mugilidae	0.0161	0.7779
	10	Lethrinidae	0.0139	0.8015
	11	Leiognathidae	0.0138	0.8250
	12	Monacanthidae	0.0136	0.8481
	13	Centrarchidae	0.0109	0.8666
	14	Pristigasteridae	0.0093	0.8824
	15	Haemulidae	0.0091	0.8980
	16	Lutjanidae	0.0080	0.9116
	17	Soleidae	0.0066	0.9228
	18	Megalopidae	0.0064	0.9336
	19	Apogonidae	0.0059	0.9437
	20	Cichlidae	0.0048	0.9518
XC2	1	Sciaenidae	0.1212	0.2494
	2	Engraulidae	0.0308	0.3128
	3	Monacanthidae	0.0292	0.3728
	4	Siganidae	0.0291	0.4328
	5	Serranidae	0.0262	0.4867
	6	Carangidae	0.0252	0.5385
	7	Haemulidae	0.0221	0.5839
	8	Scombridae	0.0221	0.6293
	9	Hemiscylliidae	0.0209	0.6723
	10	Clupeidae	0.0198	0.7130
	11	Myctophidae	0.0148	0.7434
	12	Sillaginidae	0.0109	0.7660
	13	Lethrinidae	0.0082	0.7828
	14	Leiognathidae	0.0081	0.7994
	15	Gerreidae	0.0078	0.8155
	16	Plotosidae	0.0075	0.8309
	17	Gobiidae	0.0071	0.8455
	18	Apogonidae	0.0065	0.8589
	19	Rachycentridae	0.0065	0.8723
	20	Pristigasteridae	0.0055	0.8836

XC3	1	Clupeidae	0.0556	0.1440
	2	Gerreidae	0.0351	0.2350
	3	Monacanthidae	0.0239	0.2969
	4	Mugilidae	0.0227	0.3558
	5	Rachycentridae	0.0199	0.4072
	6	Engraulidae	0.0191	0.4568
	7	Scombridae	0.0191	0.5063
	8	Ambassidae	0.0149	0.5449
	9	Leiognathidae	0.0135	0.5800
	10	Carangidae	0.0131	0.6140
	11	Haemulidae	0.0127	0.6468
	12	Siganidae	0.0105	0.6740
	13	Muraenidae	0.0093	0.6981
	14	Acropomatidae	0.0080	0.7189
	15	Terapontidae	0.0078	0.7390
	16	Gobiidae	0.0072	0.7577
	17	Lophiidae	0.0070	0.7759
	18	Synodontidae	0.0066	0.7930
	19	Mullidae	0.0065	0.8097
	20	Serranidae	0.0062	0.8257
XC4	1	Engraulidae	0.1546	0.2273
	2	Serranidae	0.1123	0.3923
	3	Siganidae	0.0666	0.4903
	4	Clupeidae	0.0499	0.5637
	5	Platycephalidae	0.0463	0.6318
	6	Mugilidae	0.0356	0.6841
	7	Sciaenidae	0.0244	0.7200
	8	Gerreidae	0.0219	0.7521
	9	Sillaginidae	0.0188	0.7798
	10	Carangidae	0.0137	0.7999
	11	Tetraodontidae	0.0122	0.8179
	12	Elopidae	0.0095	0.8318
	13	Callionymidae	0.0091	0.8452
	14	Leiognathidae	0.0089	0.8583
	15	Sphyraenidae	0.0082	0.8703
	16	Rachycentridae	0.0074	0.8812
	17	Scombridae	0.0072	0.8919
	18	Myctophidae	0.0063	0.9012
	19	Lutjanidae	0.0051	0.9087
	20	Hemiscylliidae	0.0048	0.9157
XC5	1	Clupeidae	0.1036	0.2052
	2	Engraulidae	0.0575	0.3190
	3	Sciaenidae	0.0575	0.4329

	4	Siganidae	0.0537	0.5392
	5	Serranidae	0.0448	0.6278
	6	Scombridae	0.0228	0.6729
	7	Gerreidae	0.0181	0.7086
	8	Soleidae	0.0176	0.7435
	9	Carangidae	0.0115	0.7661
	10	Pristigasteridae	0.0097	0.7853
	11	Hemiscylliidae	0.0094	0.8040
	12	Leiognathidae	0.0082	0.8203
	13	Lutjanidae	0.0078	0.8357
	14	Rachycentridae	0.0078	0.8510
	15	Acropomatidae	0.0077	0.8663
	16	Mugilidae	0.0076	0.8813
	17	Cichlidae	0.0076	0.8964
	18	Ambassidae	0.0040	0.9042
	19	Moronidae	0.0039	0.9119
	20	Monacanthidae	0.0039	0.9196
XC6	1	Clupeidae	0.0918	0.3294
	2	Engraulidae	0.0324	0.4458
	3	Scombridae	0.0310	0.5569
	4	Leiognathidae	0.0263	0.6511
	5	Rachycentridae	0.0165	0.7102
	6	Serranidae	0.0159	0.7672
	7	Pristigasteridae	0.0087	0.7983
	8	Carangidae	0.0074	0.8250
	9	Acropomatidae	0.0054	0.8442
	10	Sciaenidae	0.0038	0.8578
	11	Synodontidae	0.0037	0.8709
	12	Siganidae	0.0033	0.8826
	13	Monacanthidae	0.0032	0.8940
	14	Apogonidae	0.0032	0.9054
	15	Gerreidae	0.0028	0.9156
	16	Menidae	0.0026	0.9247
	17	Bregmacerotidae	0.0023	0.9330
	18	Sillaginidae	0.0018	0.9394
	19	Nemipteridae	0.0016	0.9452
	20	Lutjanidae	0.0016	0.9510

Table S5 Spatiotemporal distribution of Clupeidae (%).

Sites	R1	R2	R3	R4	R5	R6
XC1	82.87	64.04	34.59	9.4	10.99	11.57
XC2	4.02	4.42	2.25	5.17	6.16	11.7
XC3	2.24	24.2	28.45	22.22	20	13.05
XC4	3.67	4.85	13.18	12.84	25.92	3.28
XC5	4.2	14.89	24.2	45.16	25.09	48.04
XC6	49.59	73	39.12	55.99	28.28	50.73
