# Supplementary figures and images for: eDNA Metabarcoding Reveals Diel Connectivity Dynamics of Fish Communities in Xincun Lagoon, Hainan
Source: Animals (Basel). 2026 Jul 22;16(14):2268. doi: 10.3390/ani16142268 (PMC13403707; doi:10.3390/ani16142268)

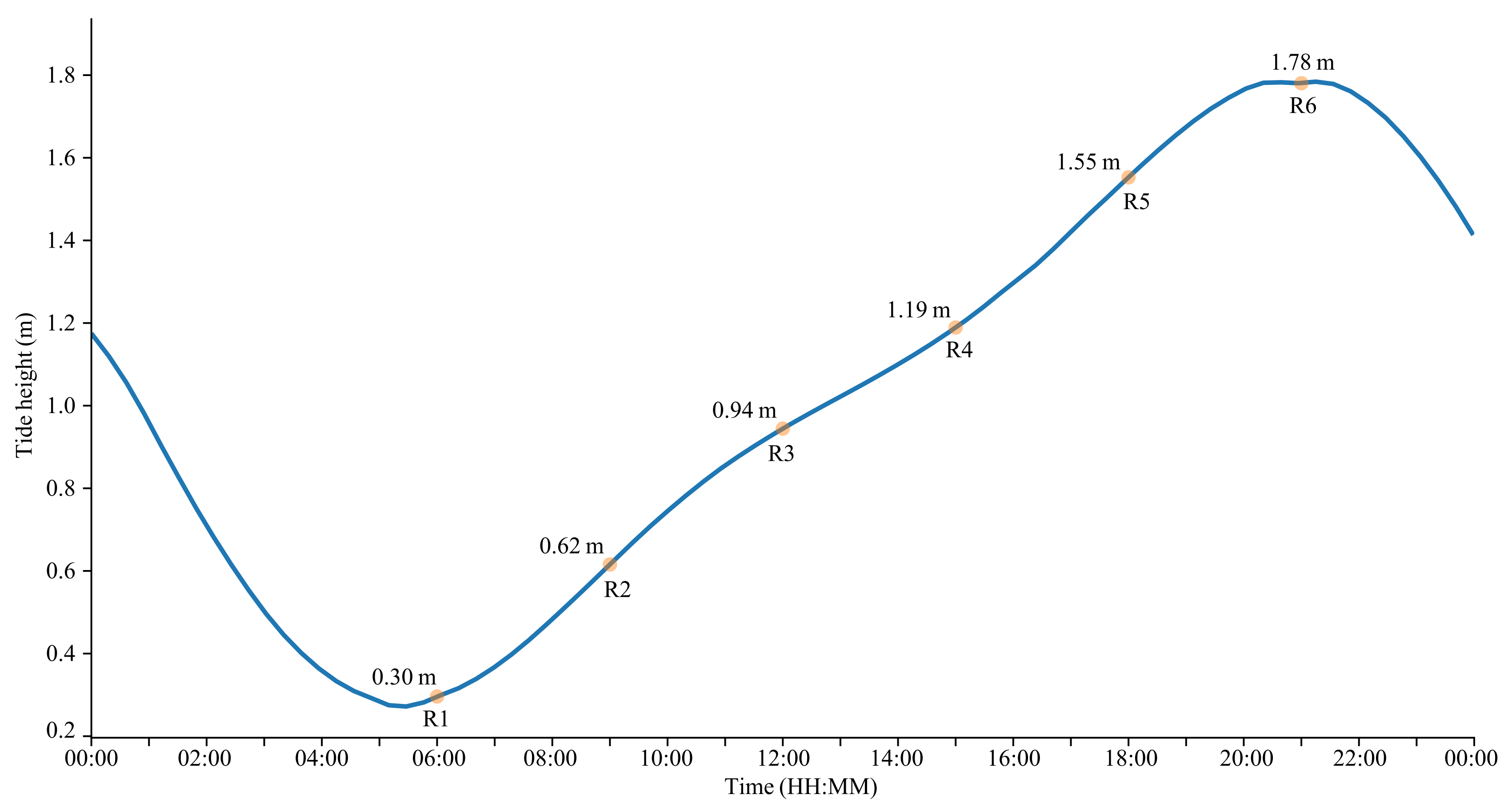

Supplement: Supplementary file 1 [file animals-16-02268-s001.zip › Figure S1. Tidal curve of Xincun Bay on January 24, 2024.pdf]
